# An Overview on the Potential Hazards of Pyrethroid Insecticides in Fish, with Special Emphasis on Cypermethrin Toxicity

**DOI:** 10.3390/ani11071880

**Published:** 2021-06-25

**Authors:** Mayada R. Farag, Mahmoud Alagawany, Rana M. Bilal, Ahmed G. A. Gewida, Kuldeep Dhama, Hany M. R. Abdel-Latif, Mahmoud S. Amer, Nallely Rivero-Perez, Adrian Zaragoza-Bastida, Yaser S. Binnaser, Gaber El-Saber Batiha, Mohammed A. E. Naiel

**Affiliations:** 1 Department of Forensic Medicine and Toxicology, Veterinary Medicine, Zagazig University, Zagazig 44511, Egypt; dr.mayadarf@gmail.com; 2 Department of Poultry, Faculty of Agriculture, Zagazig University, Zagazig 44511, Egypt; dr.mahmoud.alagwany@gmail.com; 3 Department of Animal Nutrition, Faculty of Veterinary and Animal Sciences, Baghdad ul Jadeed Campus, IUB, The Islamia University of Bahawalpur, Bahawalpur 63100, Pakistan; Muhammad.bilal@iub.edu.pk; 4 Department of Animal Production, Faculty of Agriculture, Al-Azhar University, Cairo 11884, Egypt; a.gewida@azhar.edu.eg; 5 Division of Pathology, ICAR-Indian Veterinary Research Institute, Izatnagar, Bareilly 243 122, India; kdhama@rediffmail.com; 6 Department of Poultry and Fish Diseases, Faculty of Veterinary Medicine, Alexandria University, Alexandria 22758, Egypt; hmhany@alexu.edu.eg; 7 Laser Application in Biotechnology Department, National Institute of Laser-Enhanced Science, Cairo University, Giza 12613, Egypt; m.amer@niles.edu.eg; 8 Área Académica de Medicina Veterinaria y Zootecnia, Instituto de Ciencias Agropecuaria, Universidad Autónoma del Estado de Hidalgo, Av. Universidad Km 1, Ex-Hda. de Aquetzalpa, Tulancingo 43600, Hgo, Mexico; adrian_zaragoza@uaeh.edu.mx; 9 Department of Biology, College of Sciences, Taibah University, Al-Medina Al-Munawara 41477, Saudi Arabia; yaser.binnaser@hotmail.com; 10 Department of Pharmacology and Therapeutics, Faculty of Veterinary Medicine, Damanhour University, Damanhour 22511, Egypt; gaberbatiha@gmail.com; 11 Department of Animal Production, Faculty of Agriculture, Zagazig University, Zagazig 44511, Egypt

**Keywords:** harmful effects, oxidative stress, neurotoxicity, mortalities, health

## Abstract

**Simple Summary:**

Pyrethroid insecticides are extensively used in controlling agricultural insects and treatment of ectoparasitic infestation in farm animals. However, the unhygienic disposable and seepage of pyrethroids from the agricultural runoff will lead to contamination of the aquatic ecosystems, which will, in turn, induce harmful toxic effects in the exposed living aquatic organisms, including fish. Cypermethrin (CYP) is a commonly and widely used type II pyrethroid insecticide with known dangerous toxic effects on the exposed organisms. Serious hazardous effects of these toxicants have been reported in several fish species leading to high mortalities and economic losses of the exposed fish.

**Abstract:**

Pesticides are chemicals used to control pests, such as aquatic weeds, insects, aquatic snails, and plant diseases. They are extensively used in forestry, agriculture, veterinary practices, and of great public health importance. Pesticides can be categorized according to their use into three major types (namely insecticides, herbicides, and fungicides). Water contamination by pesticides is known to induce harmful impacts on the production, reproduction, and survivability of living aquatic organisms, such as algae, aquatic plants, and fish (shellfish and finfish species). The literature and information present in this review article facilitate evaluating the toxic effects from exposure to various fish species to different concentrations of pesticides. Moreover, a brief overview of sources, classification, mechanisms of action, and toxicity signs of pyrethroid insecticides in several fish species will be illustrated with special emphasis on Cypermethrin toxicity.

## 1. Introduction

The present era of the green revolution, witnessing a swift increase in human populations across the globe, depicts the dependency of human beings on available natural resources. The current scenario has led to efforts for technological advancements to cope with the need of societies. This, in turn, is ensured by evolving ever-increasing dissolution of different synthesized chemicals in the environment, which induce pollution, specifically in aquatic bodies used as dumping sites in most parts of the world [1,2,3]. Pollution is the critical universal off-putting factor, which is worsened by the hasty growth of human populaces and rapid industrialization [4,5]. The polluted aquatic environment is a hazardous worldwide problem, and the drainage of agricultural, industrial, and commercial chemicals into the aquatic environment has induced several harmful effects on living aquatic organisms [1]. Moreover, these pollutants could directly accumulate in fish flesh and contaminate the food chain, which will consequently affect human consumers [6].

A pest refers to a rodent, insect, nematode, weed, fungus, or any other form of terrestrial or aquatic plant or animal virus, bacteria, or other microorganisms that harm the foodstuffs, garden plants, household articles, or trees, as a vector of diseases [6,7]. For farmers, pests include mites and insects that feed on crops and aquatic plants, and cause animal and plant diseases, such as fungi, viruses, bacteria, snails, nematodes, and rodents [8]. On the other hand, pesticides are referred to as many chemical compounds that possess various biological activities and chemical natures, which are clustered together to increase their capability to eradicate pests [9,10,11,12,13]. Thus, as a broad definition, pesticides are all those substances or their mixture used for prevention, destruction, repelling, deterring, resisting, or controlling pests [8,9].

Water pollution with pesticides may be due to direct application of these chemicals for controlling aquatic flora and/or seepage from agricultural lands through agricultural runoffs [14]. These are widely spread in both urban and agricultural landscapes [15]; this simply regards the pesticide residues or pesticides as major contributors to water pollution [16,17]. Across the globe, different types of pesticides are being used in different ratios, such as insecticides, which make up approximately 80% of all pesticides, herbicides (15%), and fungicides (1.46%) [9].

Pesticide residues can be sustained for long periods in the fields after application due to their decreased biodegradation properties [18], which could be absorbed by aquatic organisms, such as fish, leading to negative influences on their health and meat quality, which will negatively affect human health [7]. Furthermore, they have a quick biodegradation rate in the aquatic environment where algae and macrophytes exist [19]. These pesticides are over 100 times more poisonous for fish due to the increased sensitivity of fish to toxic agents, due to their direct contract to water via gills and absence or the insufficient hydrolytic enzymes for pyrethroids [20]. These chemicals are transformed in the hepatocytes, bile, and blood cells to sulfates and glucuronides, causing undesirable effects on meat quality and the survival rate of fish [21,22,23].

Pyrethroids are from commonly used insecticides worldwide [4,24,25,26]. Synthetic pyrethroid insecticides (such as permethrin, deltamethrin, resmethrin, tetramethrin, γ-cyhalothrin, and cypermethrin) can cause serious toxicological impacts on the exposed aquatic organisms [27]. Cypermethrin (CYP) can be defined as a fourth generation pyrethroid insecticide that is broadly used to constrain cotton pests and can also be recommended as a “pour-on treatment” to control ectoparasites of farm animals (such as ticks and mites) [28].

Studies showed that CYP induced genotoxicity and oxidative stress in the exposed zebrafish (*Danio rerio*) [29,30], malformations in rohu (*Labeo rohita*) during the early developmental stages [31], immunotoxic effects in common carp (*Cyprinus carpio*) [32], DNA damage, apoptosis, and histopathological alterations in *C. carpio* [33], hepatotoxicity in the Catla (*Catla catla*) [34], and neurotoxicity and apoptotic changes in the brain of *C. catla* [35]. Therefore, this review discusses the most toxic impacts of pesticides on fish, specifically pyrethroids, emphasizing CYP-induced toxicity.

## 2. Detrimental and Toxic Effects of Pesticides in Fish: A General Overview

Exposure to pesticides in sub-lethal and lethal doses produces toxic effects in aquatic organisms, including fish [33,36,37], which can be categorized into the following.

### 2.1. Behavioral Changes

Pesticides may induce behavioral responses, such as schooling behavior, higher mucus production from the goblet cells of the skin (sliminess), jumping, motionlessness, modification in the migration behavior, vertical (upside down) positions, sinking to the bottom, non-responsiveness with hyperexcitability, rapid, jerky movements, higher opercular rate (increased respiration rate), and changes in the body color of several fish species, such as *Tor putitora*, *C. carpio*, Mozambique tilapia (*Oreochromis mossambicus*), *L. rohita*, *C. catla*, *Cirrhinus mrigala*, *Clarias batrachus*, and *Channa punctatus* [38,39,40,41]. Moreover, they could modify and disturb the swimming behavior in aquatic vertebrates, such as fish and amphibians, and depress their growth rates [4,25]. Reports showed that exposure to pyrethroids downregulated the dopamine active transporter activity, leading to irregular behavior characteristics [42].

### 2.2. Reproductive Disorders and Malformations

Pesticides may also induce some reproductive disorders in brown trout (*Salmo trutta*) and Atlantic salmon (*Salmo salar*) [43]. Moreover, some studies reported several developmental alterations in fish exposed to the pesticide [31]. Several studies have proved the toxic effects of pyrethroids in fish reproduction and during early developmental stages. For instance, the bifenthrin and permethrin pyrethroids can delay synthesizing egg proteins (vitellogenin, choriogenin) in juvenile fish [44]. At the same trend, Wu et al. [45] stated that DLM at concentrations of 20 or 40 µg/L showed toxic effects on swim bladder development in zebrafish embryos.

### 2.3. Histopathological Alterations

Pesticides, such as malathion, carbofuran, diazinon, and dichlorvos, caused several histopathological alterations, and affected the biological functions of some vital organs such as the kidney, liver, gills, testis, and ovaries of different fish species, in the form of necrotic changes, loss of the granularity of cytoplasm, shrinkage of cells in various tissues, nuclear pycnotic alterations, vacuolation in the cytoplasm (in gill lamellae, kidneys, and filaments), degeneration of glomerulus, shrinkage of nuclear materials, ruptured epithelial lining, cytoplasm clumping, altered tubular line size, degeneration of follicular cells, collecting duct damage, and changes in ovigerous lamellae in many fish species, including *L. rohita*, *Heteropneustes fossilis*, *C. carpio*, *Channa punctatus*, *O. mossambicus*, Nile tilapia (*O. niloticus*), and *Cirrhinus mrigala* [33,46,47,48].

### 2.4. Haemato-Biochemical Alterations

Several reports demonstrated that the blood profile of fish species, such as *Tor putitora*, *L. rohita*, *O. niloticus*, *C. carpio*, *O. mossambicus*, *Channa punctatus*, rainbow trout (*Oncorhynchus mykiss*), and *C. batrachus*, may be impacted by pesticide exposure [2,49]. Furthermore, reports also explain that some well-known organophosphates, such as malathion and endosulfan, pose adverse effects on the enzyme activity, i.e., L-Keto acid-activated–glutaminase, lactate dehydrogenase (LDH) level, citrate-synthase (CS), glucose 6-phosphate phosphate dehydrogenase (G6-PDH) in the brain, liver, skeletal muscles, and the gills of *C. batrachus* and *L. rohita* [2,50,51].

### 2.5. Neurotoxicity

It was also observed that pesticides may impact acetylcholine esterase (AChE) activity, resulting in adverse effects on the nervous system of fish and, thus, produce various neurotoxic effects (neurotoxicity) [49,52]. Pesticides modified the actions of AChE in *C. carpio*, *L. rohita*, *O. mossambicus*, *Rhamdia quelen*, and *Colisa fasciatus* [53,54,55]. Furthermore, CYP-induced neurotoxicity and apoptotic changes in the brain of *C. catla* [35].

### 2.6. Endocrine Disruption

Pesticides also have an endocrine-disrupting effect on fish [56]. When used in higher concentrations, these chemical compounds may induce molecular toxicity in various types of fish, such as goldfish (*Carassius auratus*), *L. rohita* and, *Cirrhinus mrigala* [2,40,57]. In addition, histopathological studies revealed that pesticides might negatively influence the endocrine system of *L. rohita* and *O. mykiss* [58,59]. Moreover, bifenthrin has been revealed to reduce the 17-β estradiol levels in the bloodstream, consequently decreasing the ovarian follicle diameter in *O. mykiss* [60]. Moreover, bifenthrin showed higher binding capacity with thyroid hormones through the downregulation of hypothalamus-pituitary–thyroid (HPT) axis-related genes in zebrafish embryos [61].

### 2.7. Effects on Proximate Body Composition

Results of Lakshmanan et al. [62], Muthukumaravel et al. [63], and Bibi et al. [54] revealed that pesticides negatively influenced the values of proximate body composition of fish (such as crude protein, crude lipids, ash, moisture, etc.), including *O. niloticus*, *H. fossilis*, *C. batrachus*, *L. rohita*, *Colisa fasciatus*, *C. carpio*, and African catfish (*C. gariepinus*). Furthermore, a notable rise in the concentration of ascorbic acid and cholesterol in the kidney, liver, and muscles and depression in the level of glycogen, albumin, and protein contents were also recorded.

### 2.8. Oxidative Stress Injury

Exposure of fish to pesticides reduced the antioxidant defense enzyme activities, such as catalase (CAT), glutathione peroxidase (GPX), superoxide dismutase (SOD), reduced glutathione content (GSH), glutathione reductase (GR), glutathione-s-transferase (GST), and lipid peroxidation marker malondialdehyde (MDA) of *L. rohita*, *O. niloticus*, *Hoplias malabaricus*, *C. gariepinus*, *Lepomis macrochirus*, and *Tor putitora* [2,40,51,63].

### 2.9. Genotoxicity

Pesticides usage exhibit carcinogenic and genotoxic effects, which cause different forms of nuclear abnormalities, such as chromosome and chromatid breaks, centromeric attenuation, extra fragments of DNA (DNA fragmentations), pyknosis, stubbed arms besides changing the DNA replication, which leads to different kinds of mutations and cell proliferation [64]. Moreover, it was reported that increased DNA fragmentation of hepatocytes and gill cells was found in *C. carpio* exposed to sub-lethal CYP levels [33].

### 2.10. Immunotoxicity

It was reported that pesticides negatively impact the immune status of various fish species. They pose immune deficiency responses by a low level of granulocytes and lymphocytes, by inhibition of B and T cell proliferation, a decrease in phagocytic cell functions, and lower leucocytes number, which lead to reducing the resistance of fish to combat infections and diseases [65,66]. For instance, Soltanian and Fereidouni [32] clarified that chronic CYP toxicity in common carp induced immunotoxic effects, which manifested by increased mortalities after experimental challenges with pathogenic *Aeromonas hydrophila*.

The experiential changes in the above-stated factors have been found in various fish classes, including their body parts. Moreover, observations recorded in the light of the above parameters suggest the occurrence of different levels of harmful impacts for different kinds of pesticides on various tissues of exposed fish species [67].

## 3. Pyrethroid Insecticides

Pyrethroid insecticides are synthetic derivatives from pyrethrins, which some plants naturally produce, such as *Tanacetum cinerarieafolium* or *Chrysanthemum cinerariifolium* [68,69]. Chemically, the pyrethroid derives from acids and alcohols of chrysanthemum acid (ethyl 2,2-dimethyl-3-(1-isobutenyl) cyclopropane-1-carboxylate) [70,71]. Pyrethroids have been widely used for controlling insects in the agriculture and ectoparasitic infections in humans and animals [69,72,73]. Both pyrethroids and pyrethrins are highly toxic and rapidly degrade in the environment under proper temperature, light, and moisture levels [74]. The degradation process usually occurs in one or two days in sunlight and proper atmosphere. Pyrethroids are considered promising alternatives to conventional pesticides because they do not contaminate groundwater [75].

### 3.1. Classification and Types of Pyrethroids

Pyrethroids are synthetic organic insecticides divided into two distinct groups (type I and type II). Type I pyrethroids lack a cyano moiety and type II pyrethroids are with an alpha-cyano group [76]. Permethrin pesticides include bioremethrin, resmethrin, allethrin (Allyl analog), and tetramethrin, as examples of type I pyrethroids, whilst type II pyrethroids include CYP, cyphenothrin, deltamethrin (DLM), cyfluthrin, and fenvalerate. Both types of pyrethroids inhibit spontaneous activity in the neurons of target organisms [77]. Type I pyrethroids produce reflex hyperexcitation and fine tremors, while type II involves more complex syndromes, such as higher gill mucus secretions and clonic seizures. In addition, type I alters the sodium channel actions in different ways, while type II modifies transitions to the sodium channel in inactivated and open states [78].

DLM, CYP, and lambda-cyhalothrin are commonly used synthetic forms of pyrethroid insecticides worldwide. Kumar et al. [79] reported that DLM is highly effective against malaria vectors, making it efficient in the manufacture of mosquito evictor nets. It is categorized as a type II pyrethroid and is soluble in organic solvents (acetone and alcohol), but insoluble in water [80]. Besides, it is created from natural pyrethrins compounds, which quickly affect the nervous system of insects, inducing a fast knockdown influence [81]. Furthermore, DLM is linked with the prolonged opening of voltage-gated sodium channels, which causes depolarization in neuron membranes, repetitive discharges, and produced synaptic disorders [71,80]. It also diminishes the ion exchange process between chloride and calcium channels of the neurons [75,80].

CYP is an active synthetic pesticide expansively applied to households, industrial, and agricultural fields to control many insect pests. It is also categorized as a type II pyrethroid that displays stability in neutral and acidic solutions. It could prohibit the transportation process of sodium ions through the cell membrane [82].

Lambda-cyhalothrin is a synthetic acaricide pyrethroid insecticide that is used to prevent a broad spectrum of crop pests [83]. It is synthesized from a mixture of cyhalothrin isomers, which altered the nervous system functions [84]. It was restricted due to its higher toxicity to fish [85].

### 3.2. Modes of Action of Pyrethroids

Pyrethroids are categorized as neurotoxins targeting the peripheral and central nervous system axons by intermitting with sodium channels in insects [83]. In this concern, Bradberry et al. [75] reported that the selective toxin activity of pyrethroids towards insects is 2250 times higher than animals. This may be attributed to the presence of more active sodium channels and lower body temperature in insects. The toxic effects of pyrethroids in fish species, e.g., shellfish, and finfishes have been reported in several studies, and that related to the disturbing action of pyrethroids on the ion exchange process neuronal and mitochondrial membranes [86,87,88,89].

In mammals, sodium channels are the major proteins of the nervous system concerned with electrical signaling and the supporting of essential functions such as osmoregulation, heart pulse, and the activity of the brain. Some fish species, such as zebrafish, showed expression patterns of voltage-gated sodium channel genes similar to those in mammals [90]. All pyrethroid compounds caused prolonged sodium outflow with delaying in sodium activation gate closure resulting in decreased and extended sodium tail discharge [91]. Some pyrethroids increased the neurotransmitter release in the postsynaptic gap by prohibiting the calmodulin proteins responsible for connecting calcium ions and the intracellular membrane, and limiting the calcium removal process from the nerve endings, leading to reduced spontaneous neurotransmitter release [92]. The toxic effects of pyrethroids may depend on the sequences of the amino acid in sodium channels, specifically at position 918 (methionine), thereby making a difference in sensitivity [93]. Moreover, it has been shown that the toxicity of pyrethroids, such as bifenthrin, depends on the relative proportion of negative and positive pairs, with the negative pairs being more active than the positive and the neutral ones [94].

The long-term exposure to pesticides excites the outer cell membrane and the nervous system. Furthermore, some pyrethroids have an adverse effect on the γ-aminobutyric acid (GABA) receptors in the nervous filaments [27,95,96]. Moreover, they could prevent chloride ions from transportation into the nerve cells and modulate the activity of voltage-gated calcium channels [97]. There were minor preventing effects of pyrethroids towards the Ca-ATPase, Ca–Mg ATPase neurotransmitters, and the peripheral benzodiazepine receptors [98].

Pyrethroids could penetrate the epidermis and be combined directly with a carrier protein in blood or lymph. Consequently, the diffused pyrethroids and the epidermis cells directly affect the central nervous system via the connection with sensory organs of the peripheral nervous system [99]. Moreover, the pyrethroids may enter the body in a small portion through the breathing process in a vapor phase. Besides, they could penetrate the blood or hemolymph through the digestive tract during the digestion process [100].

### 3.3. Biotransformation and Acute Lethality of Pyrethroids to Fish

Several studies have documented the sensitivity of fish towards pyrethroids pesticides [95,101,102,103]. Unlike most mammals, fish are not able to produce the enzymes that hydrolysis the insecticides. The lipophilicity properties of pyrethroid compounds make them susceptible to the non-water-soluble components of the cells. They can also be quickly absorbed by the gills, even though water containing a small portion of these compounds [101]. The lethal effects of pyrethroids in fish may be due to their biotransformation properties.

Pyrethroid compounds are partly broken down in the gut by non-specific esterase, reducing their absorption rate. While in fish, many toxin levels are absorbed by gills, then rapidly enter the circulatory system [104]. In the hepatocytes, the hydrolysis of pyrethroids depends on oxidation by cytochrome P450 or carboxylesterase. It produces a high level of non-bioactive metabolites secreted via the urine and the bile [105].

It was found that the 96 h LC50 value of CYP was 27.07 μg/L on the guppy fish [74], 38.38 μg/L on *Poecilia reticulata* males [106], and 3.14 μg/L on rainbow trout [107]. Moreover, the lambda-cyhalothrin recorded 96 h LC50 of 81.83 μg/L [74], 1.6 μg/L on *C. carpio* fingerlings [84] and 1.72 μg/L for *L. rohita* [108]. For DLM, the 96 h LC50 in the guppy fishes was 31.51 μg/L [74], which doubled 15.47 μg/L or 14.9 μg/L in *O. niloticus* fingerlings [109,110]. The fish mortality percentage was depended on pesticide type, exposure time, bioavailability, mode of action, and concentration.

Pyrethroids pesticides induce different types of toxicity in fish. Some of these toxic impacts in the form of alterations in various physiological, behavioral, anatomical, biochemical, hematological, enzymatic, molecular, and hormonal aspects, are briefly illustrated in Table 1.

### 3.4. Cypermethrin as a Pyrethroid Model

Cypermethrin (CYP) [(RS)-cyano-(3-phenoxyphenyl) methyl-(IRS)-cis -Trans-3-(2, 2 dichloroethenyl)-2, 2-dimethyl-cyclopropane carboxylate], is one of extensively used and highly effective synthetic pyrethroids. It is lipophilic and synthetically obtained from a natural source known as pyrethrin. CYP is broadly engaged mainly in commercial agriculture, forestry, gardens, buildings, and farmyards to prevent and control insects [2]. CYP is used as an insecticide in a broader range of crops, such as wheat, sugarcane, brinjal, okra, cabbage, onion, lettuce, cotton, and sunflower [2,111]. Interestingly, it is thought that CYP is immobile and does not be expected to be biomagnified through the food chain.

Furthermore, CYP is commercially registered to kill soybean and cotton pests successfully [112,113]. The report of Bekele [114] suggests that proper application of this unique insecticide may effectively repel and control mosquitos, whilst the most effective results were found in preventing many kinds of malarial parasites. Moreover, globally, aquaculturists apply this insecticide to control parasitic infections, such as planktonic marine copepods [115]. Furthermore, the possible negative impacts of CYP on natural aquatic ecosystems were also reported [33,116]. CYP is the most broadly used pesticide during the past two decades in various parts of the world [117]. CYP readily enters the nervous system of the animal body and elicits cellular oxidative damage by inducing the production of free radicals and reducing the antioxidant effects of the body [118].

The study conducted by Laabs et al. [119] revealed CYP in rainwater at 0.376 µg/L concentration. The available literature is widely known and confirmed that CYP concentration is higher than the permissible range in water bodies, which can be harmful to all forms of aquatic life. Jaensson et al. [43] reported high levels of CYP in the surface water. On account of its higher lipophilicity property, it has a higher absorption rate [15]. This renders fish the most subtle, penetrating, and sensitive organism to CYP [120].

Table 2 summarizes the toxic effects of CYP in the exposed fish species. It was found that CYP exposure induced haemato-biochemical alterations in several fish species such as Nile tilapia [121], common carp [122], *Brycon amazonicus* [123], *Anabas testudineus* [124], rohu [125], *Heteropneustes fossilis* [120], *Prochilodus lineatus* [126], and *C. batrachus* [127]. Moreover, CYP induced behavioral changes in Nile tilapia [128], developmental toxicity of zebrafish [129], immunotoxicity of common carp [32], neurotoxicity of Catla [35], genotoxicity [29,30,33,130], and oxidative stress damage [131,132]. Furthermore, CYP induced serious histopathological alterations of African catfish [133], Nile tilapia [134], common carp [33], and Catla [34].

## 4. Conclusions

Our rapidly growing population requires intensification of crop production. Thus, it is essential to control pests and insects that cause economic losses in crop production. Pesticides have become a necessary part of the production cycle for eliminating plant diseases and killing pests that can drastically reduce harvestable products. Moreover, a polluted environment with a high concentration of synesthetic pyrethroids has caused several adverse effects in living aquatic organisms. This literature explained the biological modes of action of some pyrethroids in fish species and the underlying biological impacts of pyrethroid-contaminated water on reared fish. Furthermore, toxicological experiments showed individual responses against pyrethroid lethality. This review article provides insight for future research studies to evaluate the toxic effects of pyrethroid insecticides and sheds light on CYP toxicity mechanisms in fish.

## Figures and Tables

**Table 1:** Summary of toxic effects of some selected pyrethroid pesticides in some fish species.

Pyrethroids	Exposure Doses	Exposure	Fish Species	Toxic Effects	References
Bifenthrin (BF) λ-cyhalothrin (λ-CH)	1, 3, and 10 μg/L	72 h	Zebrafish (*Danio rerio*) embryos	Alterations in T4 and T3 levels (disruption of endocrine thyroid system)	[61]
Esfenvalerate	0.02, 0.2, 2 mg/L	96 h	Zebrafish (*Danio rerio*)	Acceleration hatching time exposed to 2 mg/L Behavioral changes correlated with impaired dopamine signaling	[42]
Permethrin (PM) β-cypermethrin (β-CP)	0.025, 0.125, and 0.750 μM	24 h	Zebrafish (*Danio rerio*)	Developmental toxicities, abnormal vascular development, changed locomotor activities, and thyroid disruption	[135]
Meothrin, Lambdacyhalothrin, Permethrin, Fenpropathrin, Esfenvalerate	0.0023–5.232, 0.00008–0.3465, 0.0015–0.0038, 0.0–0.0098 and 0.0053–0.2888 min–max values	--	*Mugil capito*	↑ serum creatinine and urea ↑ hepatic GSH and MDA	[136]
Deltamethrin (DLM)	CYP at 0.07, 0.014, 0.028, 0.056 μg/L	7, 14, 21 and 28 d	African catfish (*Clarias gariepinus*)	Negative effects on reproductive, biochemical, and physiological health of the exposed fish	[137]
Bifenthrin	0.5, 5, and 50 ng/L	14 and 21 d	*Menidia beryllina*	Hinder with metabolic processes and endocrine signals ↓ reproductive performance	[138]
λ-cyhalothrin	5, 50, 250, and 500 ng/L	96 h	*Prochilodus lineatus*	Oxidative stress, osmoregulatory disorders, and DNA damage	[139]
Fenvalerate EC 20%	0.92 ppm	96 h	Walking catfish (*Clarias batrachus*)	Significant damage at the hematological and biochemical levels	[140]
Beta-cyfluthrin	32, 48, 72, 180, and 450 ng/L	14 d	Rainbow trout (*Oncorhynchus mykiss*)	Impairment of feeding behavior (reduced food intake) At higher concentrations, the constant exposure led to death	[141]
Deltamethrin	15 μg/L	30 d	Nile tilapia (*Oreochromis niloticus*)	↑ CORT and GLU levels Downregulation CAT, GPX, IL-1β and IL-8 gene expressions Damage in histological structure of gills, intestine, spleen, and liver	[142]
Deltamethrin	0.25, 0.5, 1, and 2 μg /L	15 d	Zebrafish (*Danio rerio*)	Effects on aggressive behavior and swimming performances (highly neurotoxic compound)	[143]
Deltamethrin	5.2 μg /L	48 h	Zebrafish (*Danio rerio*)	Caused significant damage to the gills and liver	[144]
Deltamethrin	3 ul/L to 9 ul/L	15 d	*Cirrhinus mrigala*	Erratic swimming, hyper excitability, restlessness, difficulty in breathing, loss of equilibrium and gathering around the ventilation filter	[145]
Deltamethrin	7.33 μg/L	96 h	*Channa punctatus*	Inhibited AChE activity in brain, muscle, and gills	[146]
Deltamethrin	20 and 40 μg/L	24–96 h	Zebrafish (*Danio rerio*) embryos	Failed swim bladder inflation	[42]

Abbreviations: AChE: acetylcholinesterase, CAT: catalase, CORT: cortisol, GLU: glucose, GSH: reduced glutathione, GPX: glutathione peroxidase, IL-1β: interleukin 1 beta, IL-8: interleukin 8, MDA: malondialdehyde T3: triiodothyronine, T4: thyroxin. ↑ above arrow indicated to increase, ↓ down arrow indicating to decrease.

**Table 2:** Summary of the toxic effects of Cypermethrin (CYP) in several fish species.

Exposure Doses	Exposure Period	Fish Species	Toxic Effects	References
25, 50, 75, 100, and 125 ppm	96 h	African catfish (*Clarias gariepinus*)	Erratic movement, erosion, and hemorrhages of secondary gill lamellae Hyperplastic hepatic cells necrosis of hepatic cells in the liver tissues.	Andem et al. [133]
1.25, 2.5 µg/L	96 h	Nile tilapia (*Oreochromis niloticus*)	↓ hepatic glycogen ↓ the activities of ALP, AChE, and CAT in liver ↑ of plasma GLU level and activities of hepatic ACP, AST, and ALT	Kaviraj and Gupta [121]
	14–28 d		Anemia	
	90 d		Long-term exposure reduced the growth and deposition of protein and lipid in the body of fish	
0.22 and 0.44 µg/l	20 d	Nile tilapia (*Oreochromis niloticus*)	Histopathological alterations in gills Haemato-biochemical changes	Korkmaz et al. [134]
5.99 μg/L	96 h	Nile tilapia (*Oreochromis niloticus*)	Behavioral changes	Sarikaya [128]
0.186 ppm	35 d	Common carp (*Cyprinus carpio*)	↓ ion levels (Na+, K+ and Cl−) in blood ↓ gill Na+/K+-ATPase activity	Suvetha et al. [122]
0.4134 μg/L	30 d	Common carp (*Cyprinus carpio*)	Genotoxicity (=↑ DNA fragmentation) Histopathological alterations and apoptotic changes Hepatorenal injury	Khafaga et al. [33]
0.042, 0.085, and 0.17 μg/L	21 d	Common carp (*Cyprinus carpio*)	Immunotoxicity (=↓ LYZ activity and PA) ↑ Mortalities after challenge with *Aeromonas hydrophila*	Soltanian and Fereidouni [32]
20% of LC50	96 h	*Brycon amazonicus*	↑ liver and gill LPO 62 and 100%, respectively. ↑ SOD and CAT activities in the liver ↑ Plasma Na+, Cl− and GLU concentrations ↑ HCT, Hb and RBCs Hypertrophy and proliferation of chloride cells, blood vessels dilation, aneurysms, and hemorrhage of the lamella	de Moraes et al. [123]
0.015, 0.030, 0.045 µg /L	21 d	*Anabas testudineus*	↓ RBCs, Hb, HCT levels and thrombocyte (platelet) counts ↓ WBCs counts with the increase of CYP concentrations after 14th and 21st d	Babu Velmurugan et al. [124]
30 µg/L	5 d	Rohu (*Labeo rohita*)	↑ SOD, CAT and LPO in gills, liver, and kidney	Vijayakumar et al. [132]
1/10 and 1/50 of 96 h LC50 (The 96 h LC50 = 0.139 ppm)	45 d	Rohu (*Labeo rohita*)	Haemato-biochemical alterations	Das et al. [125]
0.124 and 0.41 µg/L	45 d	Catla (*Catla catla*)	Neurotoxicity (=↓ AChE activity in brain)	Jindal and Sharma [35]
0.21 and 0.41 μg/L	45 d	Catla (*Catla catla*)	↑ MDA and GSH content (oxidative stress) Hepatic histopathological alterations	Sharma and Jindal [34]
0.6 μg/L	9 d	Zebrafish (*Danio rerio*)	Genotoxicity of retinal cells Oxidative stress damage of retinal cells ↑ SOD and CAT activities ↑ *Sod* and *Cat* mRNA levels	Paravani et al. [30]
0.6 µg/L	9 d	Zebrafish (*Danio rerio*)	Genotoxicity of gill cells Oxidative stress damage of gill cells	Paravani et al. [29]
1 and 3 µg /L	4 or 8 d	Zebrafish (*Danio rerio*)	Hepatic oxidative stress DNA damage and apoptosis	Jin et al. [131]
0, 25, 50, 100, 200, and 400 µg/ L	96 h	Zebrafish (*Danio rerio*) embryos	Developmental toxicity	Shi et al. [129]
0.3 and 0.5 µg /L	4 h	*Heteropneustes fossilis*	↑ plasma GLU level ↓ in the level of liver glycogen ↓ ACP and ALP activities of liver ↓ ascorbic acid levels of blood, liver, and kidney	Saha and Kaviraj [120]
0.4, 0.8 and 1.2 µg/L	48 and 72 h	*Channa punctata*	Oxidative stress and genotoxicity in fish erythrocytes	Ansari et al. [130]
0.08 and 0.265 ppm	2, 4 or 8 d	*Rhamdia quelen*	Haemato-biochemical alterations	Borges et al. [147]
0.07 mg/L	10 d	*Clarias batrachus*	Inhibition in the activities of total Mg+2, and Na+-K+ATPase enzyme and glycogen content A significant induction in the levels of glycogen phosphorylase	Begum [127]
0.3 and 0.6 µg/L	2, 5 and 8 d	*Prochilodus lineatus*	↓ RBCs, Hb, HTC and MCHC values ↑ MCV and MCH values	Parma et al. [126]

Abbreviations: AChE: acetylcholinesterase, ACP: acid phosphatase, ALP: alkaline phosphatase, ALT: alanine aminotransferase, AST: aspartate aminotransferase, *Cat*: catalase gene, CAT: catalase enzyme, GLU: glucose, GPX: glutathione peroxidase, GSH: reduced glutathione, Hb: hemoglobin, HTC: hematocrit, LPO: lipid peroxidation, LYZ: lysozyme, MCH: mean corpuscular hemoglobin, MCHC: mean corpuscular hemoglobin concentration, MCV: mean corpuscular volume, MDA: malondialdehyde, PA: phagocytic activity, RBCs: red blood cells, *Sod*: superoxide dismutase gene, SOD: superoxide dismutase enzyme, WBCs: white blood cells. ↑ above arrow indicated to increase, ↓ down arrow indicating to decrease.

## Data Availability

All data sets collected and analyzed during the current study are available from the corresponding author upon fair request.
